# The role of fast and slow dynamics in nonlinear resonant ultrasound spectroscopy of consolidated granular materials

**DOI:** 10.1038/s41598-025-11854-6

**Published:** 2025-07-26

**Authors:** Jan Kober, Marco Scalerandi, Mauro Tortello, Timothy J. Ulrich, Radovan Zeman

**Affiliations:** 1 Institute of Thermomechanics of the Czech Academy of Sciences, Prague, Czechia; 2 DISAT, Condensed Matter Physics and Complex Systems Institute, Politecnico di Torino, Italy; 3 Los Alamos National Laboratory, Los Alamos, NM 87545 USA; 4 Materials Science and Engineering Dept., Texas A&M University, College Station, TX 77843 USA; 5 Faculty of Nuclear Sciences and Physical Engineering, Czech Technical University in Prague, Prague, Czechia

**Keywords:** Slow dynamics, Relaxation, Sandstones, Consolidated granular media, Modulus and damping, NRUS, Acoustics, Characterization and analytical techniques, Nonlinear phenomena

## Abstract

Elastic nonlinearity observed in consolidated granular media can be attributed to the combination of slow and fast effects, which give rise to hysteresis and relaxation of both modulus and damping after the sample is perturbed. A consequence is a high level of complexity in the measurements of the sample linear and nonlinear elastic parameters. The results of experiments are dependent on the experimental protocol that is adopted to measure the relevant quantities and it is hard to quantify parameters with accuracy and repeatability. Here we focus on examining Nonlinear Resonant Ultrasound Spectroscopy, showing experimentally the role of slow dynamics in the process and quantifying/discussing its influence on the quantification of nonlinearity. We also propose a model to describe the process, which shows that different contributions to nonlinearity (e.g., classical and hysteretic) could be due to physical features (defects) relaxing with different relaxation times.

## Introduction

The elastic nonlinearity observed in consolidated granular media can be attributed to a combination of distinct features, including fast and slow (conditioning, relaxation and creep) effects^1–5^. The interplay between these phenomena, occurring at different time scales, has been observed mainly in sandstones^6,7^, but also in other granular materials, such as mortar and concrete^8^ or metal alloys^9,10^. In some cases, experimental observations lead to results which are only partially repeatable or difficult to interpret, since the relative role of fast and slow dynamics is dependent on the measurement protocol and duration^11–13^. The major consequence is a limitation in the quantification of the nonlinear parameters, which is an issue for both development of physical models aiming to identify sources of elastic hysteresis^14–19^ and applications (e.g. in the seismic^20,21^ or in the material characterization^22–24^ domains).

Elastic hysteresis consists in a non trivial dependence of modulus and damping on strain. The observed softening of modulus and increase in damping are different when loading and unloading the sample^3,25–27^. A technique often used to evaluate material elastic moduli (modulus tensor) is the Resonant Ultrasound Spectroscopy (RUS)^28^. Since the resonant response of a sample depends on its shape and elastic constants, it is possible to compute the complete tensor of elastic moduli by inverting the frequencies of a sufficient number of resonance modes^29,30^. The easiness of experimental implementation made this technique widely used for materials characterization.

Resonant Ultrasound Spectroscopy (RUS) can be adapted to quantify the strain dependence of elastic moduli in nonlinear elastic materials. In this nonlinear regime, the goal is not to invert the full elasticity tensor, but rather to monitor how a specific modulus – typically the Young’s modulus in the case of longitudinal modes – varies with increasing strain. To this end, the measurement focuses on a single vibrational mode, tracking its resonance frequency as the drive amplitude (and thus strain) increases. The resulting technique is called Nonlinear Resonant Ultrasound Spectroscopy (NRUS)^31–34^. It is to be remarked that resonance curves allow to monitor variations of both velocity and damping, given by the shift in frequency of the resonance peak and increase in width of the resonance curve, respectively. Despite the simplicity of the experimental protocol, issues arise in the interpretation of the results^11,13,35^.

The global nature of NRUS measurements makes a comparison with local measurements necessary. Dynamic Acousto-Elastic Testing (DAET)^12,36–38^ provides an excellent tool for locally measuring velocity variations, both in time and space. Results reported in the literature indicate that to some extent NRUS and DAET observations are compatible^29^, provided anisotropy is properly taken into account^39–41^. However, we will show here that when comparing DAET and NRUS data, some contradictory results are observed, demonstrating the role of slow dynamics in NRUS.

The goal in this work is to highlight the complexity of NRUS results interpretation due to the interplay of slow and fast dynamic effects. In section 3 we will introduce an experimental protocol combining NRUS with DAET measurements and taking advantage of monitoring the baseline velocity, as proposed in other contexts^2^. Results will be analysed in section 4, discussing how cumulative conditioning effects make the definition of velocity variation critical. Also, we will introduce a possible approach to obtain an ”absolute” measurement, in the sense that results are slow dynamics independent (and thus independent from the experimental setup). Finally in section 5 a time-delayed model based on multi-relaxation^42–45^ of a distribution of non-equilibrium strain components is introduced^18,41^. Model results allow to understand the role of the different time scales in describing observations.

## Nonlinear resonant ultrasound spectroscopy and dynamic acousto-elastic testing

To introduce and motivate our work, in this section we outline briefly the main features describing velocity dependence on strain observed in experiments. Details on the experimental procedure for both NRUS and DAET are given in the next sections, when reporting our results.

### Nonlinear resonant ultrasound spectroscopy

NRUS is investigating material nonlinearity through the measurement of amplitude dependent resonance peaks. At each excitation amplitude, resonance frequency and damping are evaluated. The experimental protocol consists in injecting a sequence of continuous waves with frequency $\omega _j$ at a given input voltage amplitude $A_{i}$ and probing around one of the modes of the sample.

Experimental signals which allow constructing the resonance curves are detected from a single measurement point. From these signals the strain amplitude is usually determined. In case of bar shaped samples and longitudinal modes, the following linear assumption is adopted:

1 $$\begin{aligned} \varepsilon = \frac{V_\text {ep}}{v_\text {L}}, \end{aligned}$$

where $V_\text {ep}$ is the vibration velocity of the end of the bar and $v_\text {L}$ is the longitudinal wave velocity of the material. This is measured for each excitation frequency $\omega _j$ and amplitude $A_{i}$.

The resonance frequency can be calculated, either finding the maximum of the resonance curve using a polynomial fit or using the MoDaNE approach^46^. In both cases, $f^\text {res}_i$ (the resonance frequency at drive amplitude $A_i$) and $\varepsilon _i$ (the strain amplitude at the frequency $f=f^\text {res}_i$) are obtained. A relative frequency variation is then defined as

2 $$\begin{aligned} \delta f_i^\text {NRUS} = \frac{f^\text {res}_i - f^\text {res}_0}{f^\text {res}_0}, \end{aligned}$$

where $f_0^\text {res}$ is the resonance frequency measured at the lowest drive amplitude before beginning the experiment (linear resonance). The relative frequency variation is equivalent, as discussed later, to an effective velocity variation $\delta v_i^\text {NRUS}$.

**Figure 1:**
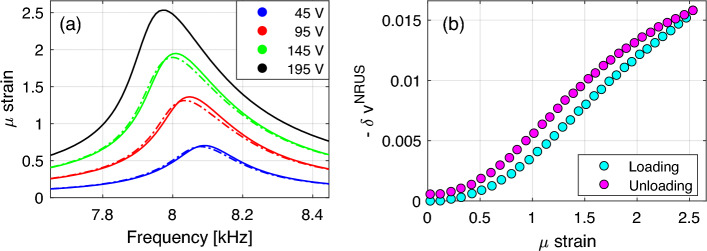
Results of a typical NRUS measurement using a sandstone sample and a sequence of successively increasing and decreasing amplitudes (loading and unloading), the protocol is described in detail section 3. (**a**) Resonance curves: output strain amplitude vs. frequency. The colors refer to different amplitudes of excitation as indicated in the plot legend. Solid lines refer to excitation at increasing amplitude; dashed lines refer to excitation at the same input amplitude, but during unloading. (**b**) Effective velocity dependence on strain during loading and unloading.

Similarly the damping relative variation could be derived, defining damping from the Q factor, e.g. from the width of the resonance curve at half height:

3 $$\begin{aligned} \delta \alpha _i^\text {NRUS} = \frac{\alpha _{i} -\alpha _0}{\alpha _0} = \frac{Q_0}{Q_i}-1, \end{aligned}$$

where $\alpha _0$ is the linear damping and $Q_0$ is the Q factor measured at low amplitude. Note that $\alpha$ here is not the hysteretic nonlinear parameter, often used in the literature.

Typical NRUS results on sandstone show decrease of the resonance frequency and increase of peak width and asymmetry with increasing drive amplitude. When the measurement protocol consists of an up-going and down-going sequence of input amplitudes $A_i$, a difference between the results for equivalent amplitudes is present both for resonance peak shape and relative frequency variation (see Fig. 1). These features can be attributed to slow dynamics^11^.

### Dynamic acousto-elastic testing

DAET experiments are conducted on bar samples in longitudinal resonance conditions. During the low frequency excitation local variations of velocity are measured from Time-of-Flight (TOF) variations of high-frequency pulses propagating along a short propagation distance (e.g. transverse direction of a sample). A transmitter sends a high frequency pulse which propagates to a receiver attached to the other side of the sample. The experiment is repeated with a given repetition rate and for each measurement time $t_i$ the output signal is detected and temporally correlated with the output signal at time $t_0$. The maximum correlation defines the variation in the TOF, which is proportional to the inverse of velocity. Therefore, the relative velocity variation is:

4 $$\begin{aligned} \delta v_i^\text {DAET} = - \frac{\Delta \text {TOF}_i}{\text {TOF}_0}, \end{aligned}$$

where $\text {TOF}_0$ is the TOF in unperturbed condition, $\Delta \text {TOF}_i$ is the TOF variation at time $t_i$. Apart from $\delta v_i^\text {DAET}$, the variation of damping coefficient can be evaluated from the peak-to-peak amplitude of received pulses $a_{\text {pp},i}$ as

5 $$\begin{aligned} \delta \alpha _{i} = -\frac{a_{\text {pp},i}-a_{\text {pp},0}}{a_{\text {pp},0}} \frac{1}{d \alpha _0}, \end{aligned}$$

where $a_{\text {pp},0}$ is the peak-to-peak amplitude in the unperturbed state, *d* is the propagation distance and $\alpha _0$ is the linear damping coefficient.

**Figure 2:**
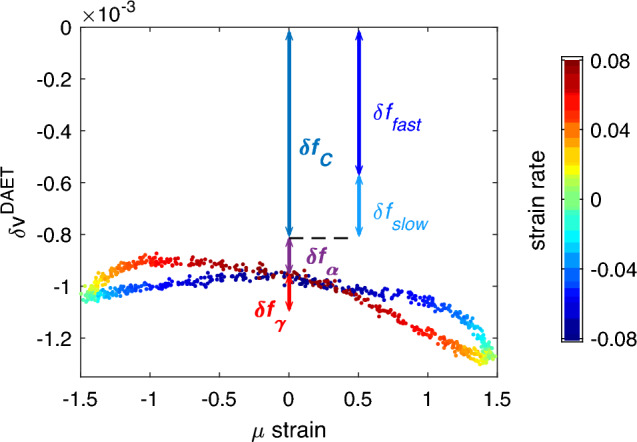
Velocity variation vs. strain obtained in a typical DAET measurement. The velocity variation can be described by components of classical and hysteretic nonlinearity and the offset (see Eq. 6). The components contributing to a frequency shift in NRUS measurements are outlined as arrows in the plot. For detailed discussion see section 2.3. This DAET measurement was conducted on a different specimen of the same source material. The sample was excited by a sinusoidal wave at frequency 8.4 kHz and amplitude 90 V. For details refer to^48^. Note that in the results reported in this paper only a few points of the hysteretic loop are probed.

DAET allows to probe velocity and damping variations at successive times while the material parameters vary due to dynamic loading, e.g. the uniaxial loading induced by the sinusoidal wave used in the NRUS experiment, which will be conducted simultaneously with DAET measurements. It is thus possible to plot $\delta v\left( t\right)$ as a function of strain. The typical result is shown in Fig. 2. Usually, the curves are phenomenologically described as the superposition of linear and quadratic components plus a hysteretic term^47^, with an additional offset:

6 $$\begin{aligned} \delta v = \beta \varepsilon + \gamma \varepsilon ^2 + \alpha \left( \Delta \varepsilon + \varepsilon {{\,\textrm{sign}\,}}{{\dot{\varepsilon }}} \right) + c. \end{aligned}$$

Here $\Delta \varepsilon$ and ${{\dot{\varepsilon }}}$ are the strain peak-to-peak amplitude and strain rate.

### Comparing NRUS and DAET observations

NRUS and DAET are probing material elastic nonlinearity differently. Where DAET allows to inspect the amplitude dependence within a single period of dynamic excitation and locally, NRUS results are averaged in time and space. Recalling the DAET results in Fig. 2, only three terms with nonzero average variations contribute to the resonance frequency variation:

7 $$\begin{aligned} \delta f = \delta f_{\gamma }+\delta f_{\alpha }+\delta f_{c}. \end{aligned}$$

The effect observed in NRUS is thus the sum of the contribution of the quadratic part of the curve $\delta f_{\gamma }$ and that due to the opening of the loops $\delta f_{\alpha }$. In addition we have a contribution $\delta f_{c}$ due to conditioning, which is known to grow increasing the duration of the applied strain (time dependent term).

We will introduce and discuss here a further decomposition of the conditioning term into two contributions: a fast and a slow part, which evolve in time on the time scale of the wave period $\left( \delta f_\text {fast}\right)$ and on a time scale of the order of minutes $\left( \delta f_\text {slow}\right)$. When stepping down from a large amplitude to a low amplitude excitation, the slow part of conditioning still continues to modify the velocity obtained from measurements taken at the low excitation (which will be referred in the following as baseline velocity). Finally, let us mention that in experiments it is usually observed that $\delta f_\alpha \sim \delta f_\gamma \ll \delta f_c$, thus velocity variations measured in NRUS mostly track conditioning effects.

Despite the simplicity of the procedure, two issues remain unsolved. The first one is related to the definition of strain amplitude, already briefly discussed before. In one-dimensional linear media strain in the center of the sample can be defined by Eq. 1 (ratio between transducers measured endpoint velocity and linear propagation velocity). When nonlinearity is present, this relation may not be correct, since the strain profile is distorted due to spatial inhomogeneity in the modulus and damping due to their strain dependence. This issue will not be further discussed here.

The second is related to the interpretation of the resonance frequency in NRUS measurements. In linear media, the resonance frequency is proportional to wave velocity, e.g. in case of the first longitudinal mode, we can evaluate the wave velocity as $v=2L f^\text {res}$, where *L* is the length of the bar. A relative change of the resonance frequency is equal to the relative change of wave velocity, i.e. $\delta f_i = \delta v_i^\text {NRUS}$. We adopt this definition, as everywhere in the literature, but this relation is generally not correct for non-classical nonlinear media for two reasons. The first is the already discussed spatial distribution of strain, which in turn creates a spatially distributed modulus change in the material. The second concerns the effects of conditioning. Each frequency sweep in NRUS provides single measurement of resonance frequency, while the real velocity is continuously varying, because for each frequency probe the strain amplitude is different (temporally and spatially dependent). In other words, NRUS provides a global measurement of material nonlinearity.

The goal of our paper is to explore the complexity of the NRUS procedure. In particular, we aim to discuss which details are lost because of averaging and discuss whether their effects on measurements are negligible and/or responsible of: a) the memory effect, consisting in different resonance curves when loading/unloading (Fig. 1a); b) the different strain dependence of velocity predicted when loading/unloading (Fig. 1b); c) the dependence of the measured velocity strain dependence on some of the experimental protocol parameters^11–13^. As a result, the comparison with DAET data allows to discuss the caveats of the nonlinearity quantification obtained using NRUS.

## Experiments and results

### Materials

We have tested several prismatic samples of Czech sandstone (with square section 25 mm $\times$ 25 mm and length 140 mm). Results for different samples are consistent and only those referring to one of them will be reported. The elastic properties of the material were determined both in longitudinal direction (using a longitudinal chirp excitation) and transverse direction (using shear and longitudinal pulse waves). The results are reported in Table 1. See^41^ for further details about the characterization procedure.

**Table 1:** Measured longitudinal and shear linear properties of the sandstone sample.

	Longitudinal	Transverse longitudinal	Transverse shear
$f \left[ \text {Hz}\right]$	${8.3} \times 10^{3}$	${1} \times 10^{6}$	${1} \times 10^{6}$
$v \left[ \text {m/s}\right]$	2540	2610	1850
$\alpha \left[ \text {Np/s}\right]$	$\approx \, 3100$	$\left( 5.7\pm 0.4\right) \times 10^5$	$\left( 1.6\pm 0.1\right) \times 10^5$

Values agree with results reported in the literature for Berea sandstone (which is very similar in composition and microstructure)^49–51^.

### Experimental setup

The sample is suspended horizontally in a styrofoam box, to reduce environmental effects due to humidity or temperature fluctuations. Also, the suspended configuration secures almost optimal free-free boundary conditions. The use of driving transducers and coupling agents follows the best practice for minimizing the nonlinear contribution of the experimental setup^52^. Preliminarily to our experiments, the same procedure was applied on a linear metal sample and results confirmed that the nonlinearity resulting from used transducers and epoxy layers was negligible compared to nonlinearity of sandstones.

**Figure 3:**
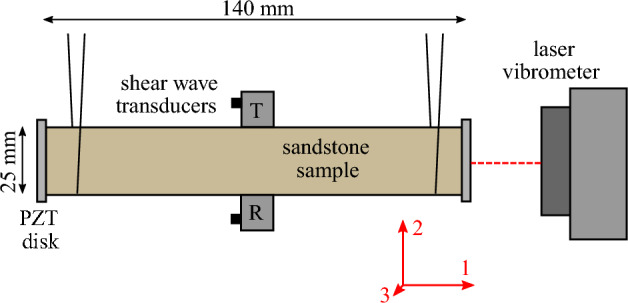
Schematic representation of the experimental setup.

The system is equipped with two pairs of transducers, each consisting of a source and a receiver, as shown in Fig. 3. Two thin low-frequency PZT rings (APC 25$\times$2.5 mm with 6 mm central hole) are glued on the bases of the prism using epoxy adhesive. One of them is used to induce high amplitude excitation in the longitudinal direction for NRUS probing. The receiver PZT disk was not used for acquisition here. The transmitter is connected to a power amplifier with amplification factor 50 (Tabor Electronics 9400). The longitudinal response is measured by a laser vibrometer (Polytec OFV-505 with OFV-5000 controller) reading the end velocity $V_\text {ep}$ in the ring PZT receiver hole. The NRUS measurement was performed with a TiePie HS5 oscilloscope-generator.

A pair of high-frequency transducers (Olympus V154, normal incidence shear wave probes) is attached in the center of the sample using a thermoplastic monomer (Crystalbond 555) so that the polarization of the waves aligns with the longitudinal direction of the sample. These are used to perform DAET measurements in the transverse direction. Keysight/Signadyne M3300A arbitrary waveform generator and oscilloscope is used for signal generation and data acquisition (14 bit resolution, 100 MSa/s).

### Experimental procedure

The experimental setup allows to simultaneously perform NRUS and DAET measurements, so that local and global velocity changes can be tracked as a function of time and strain during the entire duration of the experiment. The measurements of NRUS and DAET are synchronized but both measurement procedures run independently. The corresponding measurements of velocity variations will be labeled as $\delta v^\text {DAET}$ and $\delta v^\text {NRUS}$, respectively.

DAET measurements are performed using high-frequency transducers repeatedly sending 1 MHz pulses, with a time lag between successive pulses of 8 ms. The timing was chosen so that the pulse duration is smaller than the time-of-flight and that there is no overlap between successive probing. Velocity and damping variations are calculated using Eq. 4 and 5. Therefore, we monitor parameters variation with a temporal resolution of 8 ms.

The NRUS measurements were performed using sine-train probing in discrete frequency and amplitude steps. For each drive amplitude $A_i$, the measurement of the complete set of frequencies $f_j$ is completed before advancing in the amplitude sequence. The usual protocol is further modified to allow tracking of the linear response by alternating between high excitation amplitude $A_i$ and low (baseline) amplitude $A_\text {B}$ for each frequency step. Specifically, each measurement at input amplitude $A_i$ and frequency $f_j$ is followed by a baseline amplitude measurement at the same frequency.

The NRUS measurement parameters were determined optimally considering the sample properties and used equipment. The set of probing frequencies $f_j$ was chosen to sample the first longitudinal resonance in the range ${7.89}\,\text {kHz} \le f_j \le {8.73}\,\text {kHz}$ with a frequency step $\Delta f = {10}\,\text {Hz}$. The duration of each sine-train $\Delta t = {50}\,\text {ms}$ ensured reaching a standing wave condition. In the evaluation, the initial transient part of the response was omitted, evaluating only the last ${20}\,\text {ms}$ of the vibrometer signal by homodyne processing. The measurement is converted to strain using Eq. 1. We will label $\varepsilon _\text {C}\left( i,j\right)$ the conditioning strain corresponding to input amplitude $A_i$, and the baseline strain $\varepsilon _\text {B}\left( i,j\right)$, both at frequency $f_j$.

**Figure 4:**
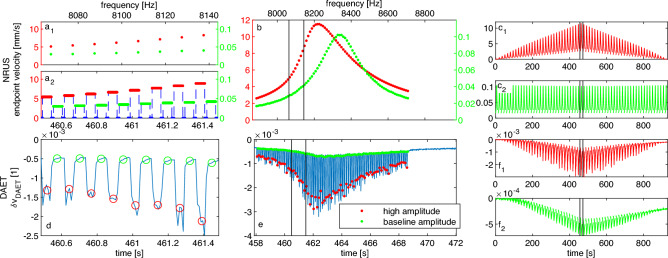
Overview of NRUS (upper row) and DAET (lower row) measurements. The whole experiment is shown on the right, while most detailed views (zooms) are reported in the central and left plots. For NRUS data, the endpoint vibration velocity is plotted vs. time or frequency. For DAET plots, the relative propagation velocity change in time is shown. The NRUS measurement procedure is represented in panels a_1_ and a_2_: at given frequency, the baseline amplitude measurement always follows the high amplitude measurement. Corresponding DAET data in panel d show alternating conditioning and relaxation phases. Since DAET is sampled faster than NRUS, averaging is performed to pair DAET and NRUS data as indicated by overlaid markers (averaging 6-7 DAET points for each frequency). Panels b and e expand the observed time window to show the distortion of resonance peak at high excitation amplitude and corresponding evolution of velocity change. Vertical black lines denote the subset shown in panels a_1,2_ and d. Panels c_1,2_ and f_1,2_ show the entire experiment timeline for NRUS and DAET data. Again, vertical black lines denote the subset shown in panels b and e. Note the cumulative effects in DAET baseline measurements in panel f_2_, but also, less pronounced, in the high amplitude ones, in panel f_2_.

The delay between successive probings (at successive frequencies) was ${12}\,\text {ms}$. After each frequency sweep, before moving to the successive amplitude, a longer ${3}\,\text {s}$ pause follows, during which data collection occurs. Each sweep is repeated at different input amplitudes $A_i$ from $A_1 = {15}\,\text {V}$ to a maximum amplitude $A_\text {max} = {195}\,\text {V}$ (loading phase) and then back to $A_1$ (unloading phase) in 63 equal steps. The baseline amplitude is $A_\text {B}=A_1$.

### Results

#### Experimental timeline

The raw results of the experiment are shown in Fig. 4. Here, the measurements are plotted mostly as a function of time, which is crucial in understanding the response of a material exhibiting slow dynamic response. First, let us consider a zoom on a very short experimental time window, with fixed drive amplitude and including only few (specifically 9) testing frequencies. The view in panel Fig. 4a explains the relation between time and frequency axes in the alternating NRUS amplitude protocol (each time corresponds to a frequency). The corresponding DAET measurements in Fig. 4d show the development of relative velocity change at a short timescale. The successive 7-8 acquisition points (at given amplitude and frequency) indicate that NRUS probing results in a sequence of partial conditioning and incomplete relaxation phases.

In panels Fig. 4b,e, is the observed time window expanded to include the duration of a single NRUS frequency sweep (at a given drive amplitude). The high amplitude NRUS peak in Fig. 4b shows a clear resonance frequency shift and a distortion of its shape with respect to the baseline amplitude measurement. Note that in this plot, both time and frequency axes are (approximately) applicable. This means that the difference between high amplitude and baseline resonant frequencies causes a time-lag between reaching these resonances, which can be as high as ${1.5}\,\text {s}$. Sweeping over the resonance peak in NRUS, results in significant changes in conditioning strain amplitudes. As evidenced in Fig. 4e, the elastic properties of the material are evolving constantly with partial memory tracked by baseline DAET measurements.

In order to complete the overview of the experimental timeline, panels c and f in Fig. 4 show the whole duration of the experiment. Notable is especially the evolution of baseline velocity change, where the effects of cumulative conditioning are the cause of incomplete recovery to the original linear state. The role of such a cumulative conditioning and its influence on results is discussed in the next section.

#### Variables

The protocol described above allows to measure strain, velocity and damping variations for each input amplitude and frequency, both at baseline excitation amplitude and conditioning excitation amplitude. For further discussion it will be useful to segment the timeline data according to conditioning input amplitudes $A_i$ and frequencies $f_j$. The variables measured in the experiment form the following matrices:

- $\varepsilon _\text {C}(i,j)$ and $\varepsilon _\text {B}\left( i,j\right)$: the strain amplitude matrices (conditioning and baseline);
- $\delta v_\text {C}^\text {DAET}(i,j)$ and $\delta v_\text {B}^\text {DAET}\left( i,j\right)$: the relative velocity variation matrices (conditioned and baseline);
- $\delta \alpha _\text {C}^\text {DAET}\left( i,j\right)$ and $\delta \alpha _\text {B}^\text {DAET}\left( i,j\right)$: the relative damping variation matrices (conditioned and baseline);

Each element of the matrix corresponds to a given acquisition time $t^\text {acq}(i,j)$.

Furthermore, from the resonance curves at each amplitude (strain vs. frequency) the resonance frequencies and Q factors can be derived and the NRUS measured velocity variations are also available: $\delta v_\text {C}^\text {NRUS}(i)$ and $\delta v_\text {B}^\text {NRUS}(i)$ (conditioned and baseline). Similar notation is used for NRUS damping relative variations.

## Discussion

In this section we focus on the results obtained for the velocity variation. Similar conclusions can be drawn for the damping variations, as discussed in supplementary material A.

### Frequency dependence

#### Definition of velocity variation at each amplitude of excitation

**Figure 5:**
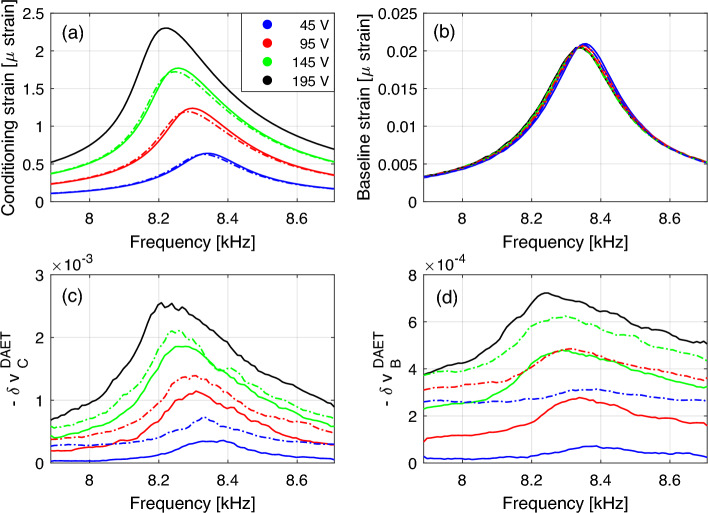
Frequency dependence. (**a**) Conditioning strain amplitude ($\varepsilon _\text {C}$) vs. frequency. (**b**) Baseline strain amplitude ($\varepsilon _\text {B}$) vs. frequency. (**c**) Relative velocity variation vs. frequency when exciting at the conditioning strain. (**d**) Relative velocity variation vs. frequency when exciting at the baseline strain. Solid/dashed lines refer to results during loading/unloading. Different colors indicate different excitation amplitudes given in the plot legend.

For each excitation amplitude, strain and velocity variation dependence on frequency can be drawn, as shown in Fig. 5. The conditioning strain dependence on frequency is that expected in any NRUS measurement (compare Fig. 5a to Fig. 1). The behavior of the baseline strain is also consistent with other measurements: slow dynamics is responsible for the softening and damping increase observed at constant (low) excitation amplitude (Fig. 5b). Consistently with the temporal evolution results (Fig. 4c), the effect of cumulative conditioning on strain is hardly noticeable, even though the hysteresis (difference between curves at loading/unloading is an indication of slow dynamic effects).

Baseline (measured while exciting at baseline strain) and conditioning (measured while exciting at conditioning strain) velocity variations follow similar profiles as a function of frequency as strain profiles (Fig. 5cd). Hysteresis (difference between loading and unloading branches) is more remarked, particularly for baseline velocity (compare solid with dashed line curves of the same colour). Effects of cumulative conditioning are present.

The results reported allow to define, for each amplitude of the excitation (each curve) the corresponding relative velocity variation. In NRUS measurements, it is given as the relative resonance frequency variation (shift in frequency of the resonance peak measurable from the upper plots of Fig. 5) and due to hysteresis is different in the loading unloading branches (giving rise to hysteretic loops as shown in Fig. 1b). Several (one per frequency) DAET measured relative velocity variations are measured for a single conditioning input amplitude. These values represent the actual (local in time and space) dependence of velocity on strain. More discussion about this issue is given in the next subsections.

#### Delay between maximum strain and maximum velocity variation

**Figure 6:**
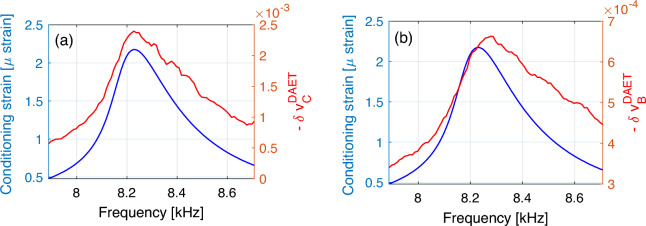
Frequency dependence. Conditioning strain amplitude and velocity variations vs. frequency for a selected input amplitude. (**a**) Conditioned velocity variation. (**b**) Baseline velocity variation. Observe the shift of the maxima of blue and red curves. Note that the blue curve is the same in both plots and represents the resonance peak for excitation amplitude of 182.5 V. For reference, the corresponding $\delta v_\text {C}^\text {NRUS} = {1.47} \times 10^{-3}$. The larger effect of velocity in NRUS measurements compared to DAET is due to anisotropy effects and is consistent with previous works^18^.

In Fig. 6, for a given conditioning input amplitude, conditioning output strain and velocity variation are plotted vs. frequency, for both conditioned and baseline measurements. There is a shift between resonance frequency (position of the peak of the blue curve) and the frequency corresponding to the maximum velocity variation (position of the peak of the red curve). The effect (clearly visible for baseline measurements and slightly for conditioning measurements) indicates cumulative conditioning: when conditioning strain amplitude already diminishes after reaching the resonance peak, the velocity still decreases (material still softens). This phenomenon is not captured by NRUS measurements. Fig. 6 shows typical response of the investigated material. The delay of maxima for both the conditioning and the baseline velocity variation with respect to maximum of strain, was investigated, but it did not show a clear dependence on driving amplitude. Similar results are also observed for damping variation as a function of frequency, as shown in supplementary material A.

### Conditioning strain amplitude dependence

**Figure 7:**
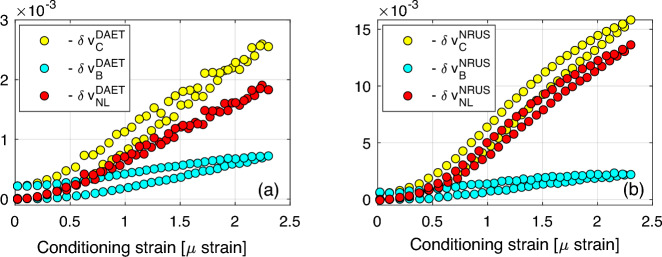
Strain dependence. (**a**) DAET relative velocity variation vs. conditioning strain. (**b**) NRUS relative velocity variation vs. strain. In both subplots, baseline and conditioned curves are subtracted to obtain the fast dynamics contribution to the measurement. We recall that, as discussed in the text, $\delta v_\text{NL}$ represents the repeatable contributions of the quadratic and hysteretic terms and fast conditioning.

The strain amplitude dependence of the relative velocity variation is discussed in Fig. 7. As already remarked, for each drive amplitude, both conditioning strain amplitude and DAET measured relative velocity variation depend on frequency (Fig. 5). Thus, we define the strain and velocity variations at drive amplitude $A_i$ as the strain and relative velocity variations at the resonance frequency (frequency corresponding to the maximum of the curves in Fig. 6). Defining $j^\text {res}$ the index at which $\omega _j=\omega ^\text {res}$, we introduce the quantities

8 $$\begin{aligned} {\tilde{\varepsilon }}\left( i\right)= & \varepsilon _\text {C}\left( i,j^\text {res}\right) , \nonumber \\ \delta {\tilde{v}}_\text {C,B}^\text {DAET} \left( i\right)= & \delta v_\text {C,B} ^\text {DAET}\left( i,j^\text {res}\right) , \end{aligned}$$

for both baseline and conditioning.

In Fig. 7a, $\delta {\tilde{v}}_\text {C,B}^\text {DAET} \left( i\right)$ is plotted vs. ${\tilde{\varepsilon }}\left( i\right)$ (i.e. conditioning strain at resonance). Each point refers to a different input amplitude. Hysteresis is observed for both conditioned and baseline data. Subtracting baseline from conditioned data should result in the elimination of the cumulative slow dynamics contribution to the measured velocity variation, preserving the repeatable contributions of the quadratic and hysteretic terms and fast conditioning (see Section 2.3). Defining

9 $$\begin{aligned} \delta {\tilde{v}}_\text {NL}^\text {DAET} = \delta {\tilde{v}}_\text {C}^\text {DAET}-\delta {\tilde{v}}_\text {B}^\text {DAET}, \end{aligned}$$

hysteresis disappears almost completely: see Fig. 7a.

Results obtained using NRUS estimates of velocity are also reported in Fig. 7b and look as expected from other works: an initial quadratic behavior, followed by a linear dependence and a final bending (in most experiments found in the literature strain ranges used do not allow to appreciate the last phase of the curve). However, it is evident, particularly at large strains, that the strain dependence predicted in NRUS is different (not only quantitatively) with respect to the one predicted using DAET data. Also, slow dynamic effects cannot be completely removed when subtracting baseline data (defining $\delta v^\text {NRUS}_\text {NL}$ analogously to Eq. 9) – a hysteresis residual loop is still present. Reasons for the slight discrepancy in the strain dependence predicted by DAET and NRUS, is that NRUS provides a global measurement of velocity, which is sensitive to the spatial strain profile. The latter could vary with strain amplitude, considering the huge variation of damping at different strains (up to 10 percent), with a consequent influence on the observed strain dependence on velocity in NRUS measurements. Correcting the influence of the strain profile is thus required and assuming the strain in the centre of the sample (where DAET measurements are taken) is proportional to the amplitude of the vibration velocity measured at the edge might be not sufficiently robust for a correct interpretation of the data (work in progress). A partial confirmation of the role of distortion at high amplitude in the spatial distribution of strain comes from anisotropy (see supplementary material B). Additional results concerning the velocity variation strain dependence are discussed in supplementary material C.

## Theory

### Non-equilibrium strain model

The first-order acousto-elastic model^53^ defines the velocity variation as proportional to strain through the first-order acousto-elastic coefficient $\beta$. We assume that the real strain in the material is the sum of the propagating strain $\varepsilon$ and a non-equilibrium strain $\varepsilon _\text {neq}$, which could be considered as the hysteretic equivalent of the plastic strain in plasticity theories (see^41^). It follows:

10 $$\begin{aligned} \delta v = \beta \left( \varepsilon +\varepsilon _\text {neq}\right) , \end{aligned}$$

where $\beta$ is the coefficient of classical nonlinearity. Indices (omitted here) should be considered to account for anisotropy depending on waves propagation and polarisation directions.

The evolution of velocity with strain is a multi-relaxation process^43,44^. To account for it, we define $\varepsilon _\text {neq}$ as the superposition of components with different relaxation times $\tau$ with a distribution $F\left( \tau \right)$:

11 $$\begin{aligned} \varepsilon _\text {neq}=\int _{0}^{+\infty }F\left( \tau \right) \varepsilon _\text {neq}^{\tau } \textrm{d} \tau . \end{aligned}$$

In analogy with what done elsewhere^19^, we choose

12 $$\begin{aligned} F\left( \tau \right) ={\left\{ \begin{array}{ll}\frac{1}{\tau ^k} & \quad \tau _\textrm{min}<\tau <\tau _\textrm{max}, \\ 0 & \quad \text {elsewhere.}\end{array}\right. } \end{aligned}$$

The choice of $F(\tau )$ is not unique, provided it satisfies some basic properties (presence of a maximum and asymmetry)^45^, and Eq. 12 might be considered also as an approximation of the generalised Weibull distribution function used elsewhere^44^. Here *k* is a positive constant parameter.

The non-equilibrium strain $\varepsilon _\text {neq}^{\tau }$ is a function of time and its evolution results from the combination of a conditioning term and a relaxation term:

13 $$\begin{aligned} \frac{\textrm{d} \varepsilon _\text {neq}^{\tau }}{\textrm{d}t} = \frac{\lambda }{\tau } A_\varepsilon - \frac{1}{\tau } \varepsilon _\text {neq}^{\tau }, \end{aligned}$$

where $\lambda$ is a positive hysteretic parameter and $A_\varepsilon$ the strain amplitude. Eq. 13 is an approximation, since the conditioning term should be proportional to strain (or more generally to a function of strain and/or strain rate), which is actually evolving in time, rather than to strain amplitude. However, the approximation is reasonable, considering that in NRUS experiment the strain is a sinusoidal function in steady state conditions and the procedure averages over one (or more) wave period.

If the strain amplitude $A_\varepsilon$ is constant, the solution of Eq. 13 is

14 $$\begin{aligned} \varepsilon _\text {neq}^{\tau }\left( t\right) =\lambda A_\varepsilon \left( 1-e^{-t/\tau }\right) + \varepsilon _{\text {neq},0}^{\tau } e^{-t/\tau }, \end{aligned}$$

where $\varepsilon _{\text {neq},0}^{\tau }$ is the initial condition. When considering a sequence of conditioning strains with amplitudes $A_{\varepsilon ,i}$ each of the same duration $\Delta t$, Eq. 14 allows to obtain the solution piecewise, i.e. as a sequence of solutions in the given form, each with a different initial condition and calculated in the time interval $0\le t \le \Delta t$. Therefore the solution includes memory of the strain history, giving rise to a cumulative effect caused by the past conditioning events and included in the relaxation of the initial condition term.

In the following subsections, we present results obtained with $k=0.95$, $\beta =100$ and $\lambda =0.1$. Note that only the product $\beta \lambda =10$ is relevant.

### Contribution to cumulative effects of various components

**Figure 8:**
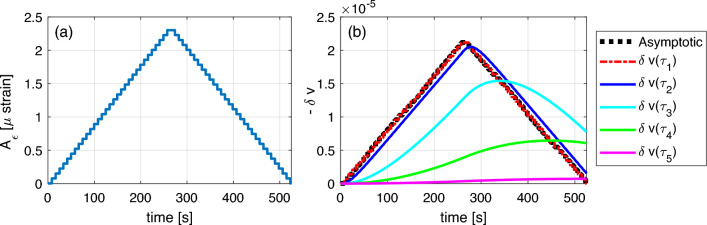
Contribution of different components to the cumulative effects. (**a**) Loading protocol. (**b**) Evolution of non-equilibrium velocity components with different relaxation times. The black curves (superimposed to the red one) represent the contribution for an infinite duration of each conditioning strain amplitude (asymptotic behavior). Coloured lines represent the solution for increasing values of $\tau$.

The contribution of the various components to the cumulative effect depends on the relaxation time $\tau$: fast $\tau$ components give little or no contribution, while slow ones play a role. Let us consider a simple excitation protocol based on a sequence of monochromatic waves at fixed frequency: the strain amplitude first increases monotonically as a function of time and then starts decreasing at the same rate. Each strain amplitude is kept on for a time interval $\Delta t={4}\,\text {s}$ (which is the duration in the experiments of a sweep over frequency at a given drive amplitude). Fig. 8 reports, for five different relaxation times, both the actual contribution to velocity variation $\delta v^\tau =\beta \varepsilon _\text {neq}^{\tau }\left( t\right)$ and the asymptotic one $\delta v^\tau _\text {asymptotic}=\beta \lambda A_\varepsilon$, i.e., the value of relative velocity variation that would be reached for $\Delta t \rightarrow \infty$ (see Eq. 14) . Recall that these quantities, weighted by $F(\tau )$, are integrated to obtain the velocity variation.

Asymptotic values for all $\tau$s are reported as black lines. They are $\tau$-independent and indistinguishable from the behavior of the fastest relaxation component (red line). The larger $\tau$ is, the stronger is the difference between asymptotic and real contribution to the velocity variation. Note that $\delta v^\tau$ starts decreasing as a function of time when it has reached its corresponding asymptotic value.

### Comparison with experiments

**Figure 9:**
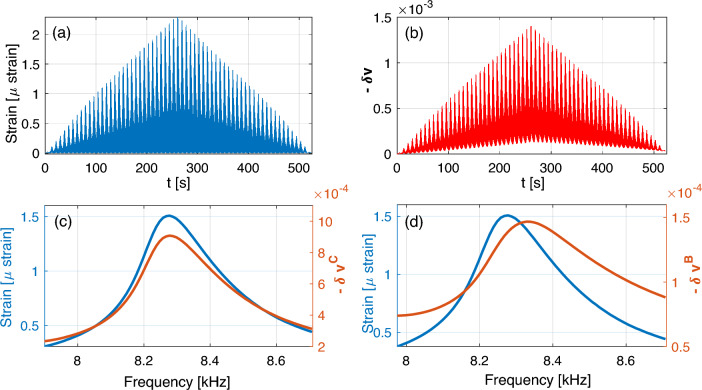
Simulation of the experiment. (**a**) Strain vs. time. (**b**) Velocity variation vs. time, to be compared with results in Fig. 4c. (**c**) and (**d**) Strain (blue line, $A_\varepsilon$, input of the model) and conditioning and baseline velocity variations (in the two subplots) vs. frequency for a selected conditioning amplitude (compare with Fig. 6).

The proposed model gives a solution for experiments with an arbitrary amplitude $A_\varepsilon$ protocol and duration $\Delta t$. We obtain the model prediction using for $A_\varepsilon \left( t\right)$ the experimental protocol for the strain vs. time reported in Fig. 4c, which was derived in experiments. The protocol is shown again in Fig. 9a. The result obtained using the model is reported in Fig. 9b. Note that it is possible to reproduce the cumulative effect.

Moreover, Fig. 9cd shows the model results reproducing the experimental ones reported in Fig. 6: also in this case we can notice a shift, much more visible in the case of the baseline, between the resonance frequency and the maximum relative velocity variation, due to cumulative conditioning, as already discussed before.

**Figure 10:**
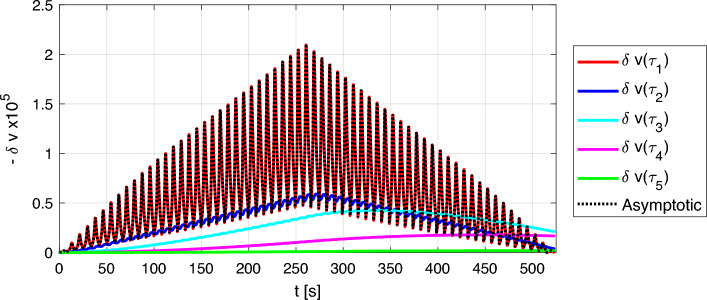
Contributions of different non-equilibrium components to the temporal evolution of velocity.

Results reported in Fig. 9 could be further interpreted by looking at the contribution to the relative velocity variation by components of the non-equilibrium strain for different $\tau$s. Similarly to what is shown in Fig. 8, in Fig. 10, we compare the actual $\delta v^\tau$ with the corresponding asymptotic behavior, reported as black lines. The model allows us to classify contributions into two classes, corresponding to what is normally defined as fast and slow dynamics:

- Components with fast relaxation times contribute to the velocity variations with an oscillating term that perfectly matches the oscillation of the strain, being the match weaker when $\tau$ increases. Conditioning and relaxation times are shorter/same order as the wave period.
- Components with slow relaxation times give a monotonously increasing contribution to velocity variations, delayed with respect to strain because relaxation times are too slow to follow the strain oscillations within one/few periods. Once reached the asymptotic conditioned value, their contribution decreases slowly. These components are responsible of cumulative effects.

## Conclusions

We have investigated the complexity hidden in the interpretation of the velocity variations measured by NRUS at different amplitudes of excitation. By comparing NRUS results with local measurements of velocity variation obtained using DAET, we have shown that the strain dependence of the predicted local velocity does not match that predicted in NRUS, particularly at large strains. This is a consequence of a cumulative conditioning effect that is evident in our experiments and can be justified theoretically by introducing a simple multi-relaxation model, which has proven to be efficient in describing the relaxation of linear velocity from a perturbed (conditioned) value to the equilibrium (linear) value.

Our work allows for some general conclusions that impact both theoretical aspects and applications:

- Both model results and experiments suggest that different time scales contribute to the definition of different features of the nonlinear dependence on strain. In other words, the result of each measurement is a combination of fast and slow features. The latter are responsible for the observed hysteresis (Fig. 1a), which is often called the memory effect. In local measurements (DAET) the slow components could be removed more efficiently, allowing to analyze fast dynamics.
- While in global measurements cumulative conditioning could not be monitored (since NRUS measurements are obtained averaging over times of the order of several wave periods), the effects of a delay in conditioning (i.e. the sample still conditions while the strain amplitude already decreases) could be easily observed and quantified (see Fig. 6).
- Cumulative conditioning makes it critical to assess quantitatively experimental results and could provide a simple explanation why the strain dependence on velocity observed in the lab depends on some of the experimental protocol parameters^11–13^.
- The caveats on the use of NRUS as an estimator of the nonlinear response of a material have impacts for applications as well. Indeed, slow dynamics is observed in sandstones, as discussed here, but also in rocks, concrete and micro-cracked metallic and composites samples. For instance, observations of slow dynamics are fundamental in the study of the evolution of the properties of the earth crust after earthquakes or other stress fields. In the field of Nondestructive Testing (NDT) the hysteresis in the elastic response is manifested at very low strains and for damage states which are often invisible to linear ultrasonic NDT. However, quantitative nonlinear NDT should carefully take into account the cumulative conditioning effects discussed here, which are normally neglected, thus making it difficult to reliably compare results obtained in different experimental conditions.

Most likely, cumulative conditioning might contribute significantly also to influence the evolution in time of the strain spatial profile in the sample during the experiment. It follows that the choice of estimating the strain as proportional to the vibration velocity detected at the end of the bar, independently of the amplitude of the excitation, is not sufficiently robust/accurate. This might contribute to explain the slight difference in the functional dependence of velocity on strain measured using DAET and NRUS.

Further work is however needed. From the theoretical point of view, the model proposed here captures well the behavior of the offset in DAET experiments. However it is too simple to capture the full phenomenology and an alternative to Eq. 13 must be developed, introducing the actual strain (and its time derivative) instead of the strain amplitude, without loosing the features manifested by the present model. On the other hand, understanding the role of the strain profile in determining correctly the dependence on strain of NRUS measured velocities is in our opinion a priority. Also, clearly identifying the physical sources of the competition between conditioning and relaxation observed in this work (see Eq. 13) is fundamental, particularly in view of applications^48^.

## Supplementary Information

Supplementary Information.

## Data Availability

Data sets generated during the current study are available from the corresponding author on reasonable request.
