# Supplementary material for: The role of fast and slow dynamics in nonlinear resonant ultrasound spectroscopy of consolidated granular materials
Source: Sci Rep. 2025 Jul 26;15:27286. doi: 10.1038/s41598-025-11854-6 (PMC12297323; doi:10.1038/s41598-025-11854-6)
Supplement: Supplementary file 1 — Supplementary Information. [file 41598_2025_11854_MOESM1_ESM.pdf]

Supplementary Information: The Role of Fast and Slow Dynamics in Nonlinear Resonant Ultrasound Spectroscopy of Consolidated Granular Materials

Jan Kober[1,*], Marco Scalerandi[2], Mauro Tortello[2], Timothy J. Ulrich[3,4], and Radovan Zeman[1,5]

[1]Institute of Thermomechanics of the Czech Academy of Sciences, Prague, Czechia
[2]DISAT, Condensed Matter Physics and Complex Systems Institute, Politecnico di Torino, Italy
[3]Los Alamos National Laboratory, Los Alamos, NM, 87545, USA
[4]Materials Science and Engineering Dept., Texas A&M University, College Station, Texas, 77843, USA
[5]Faculty of Nuclear Sciences and Physical Engineering, Czech Technical University in Prague, Czechia
[*]kober@it.cas.cz

A Damping dependence on strain and frequency

The very same analysis reported to discuss the behavior of relative velocity variations (Fig. 5 and 7) can be performed by analysing damping variations, defined in Eq. 3 and 5, for NRUS ad DAET measurements, respectively. Their temporal evolution during the experiment (shown in Fig. A.1) indicates a cumulative conditioning effect. Results for damping and velocity are very similar.

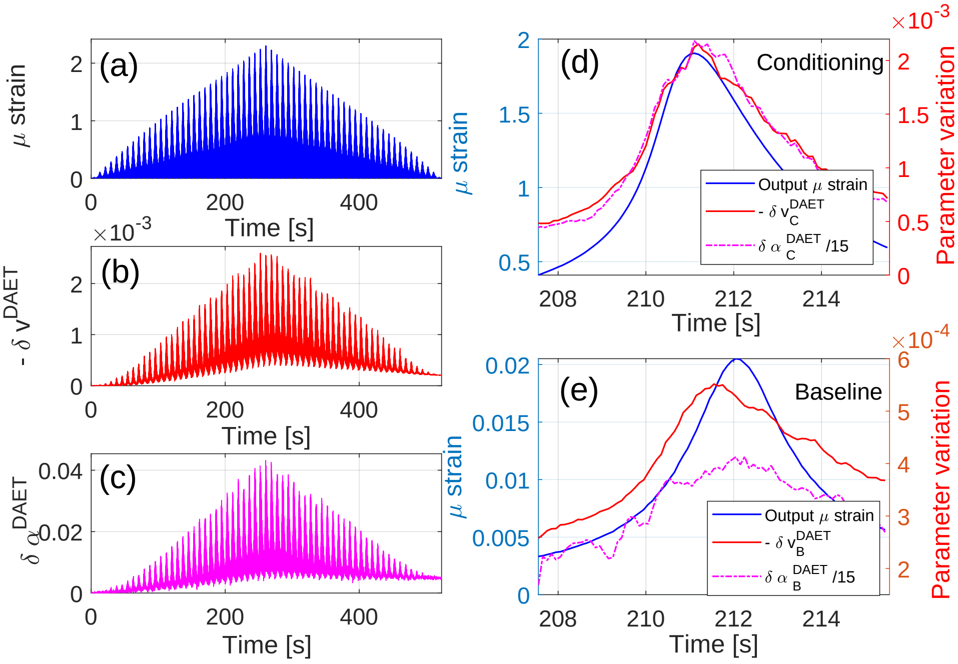

Figure A.1. Temporal evolution of strain, velocity and damping variations. In the right column a zoom over a time window corresponding to a single frequency sweep at constant excitation amplitude is reported, separating results for conditioning and baseline measurements in the two subplots.

As shown in Fig. A.2, the behavior of damping variation vs. frequency is fully consistent with the results for velocity (see Fig. 6). Results are more noisy, but the delay between maximum strain and the time at which maximum damping variations occur (lower row) is even larger than for velocity and clearly evident also for the conditioned data (Fig. A.2c).

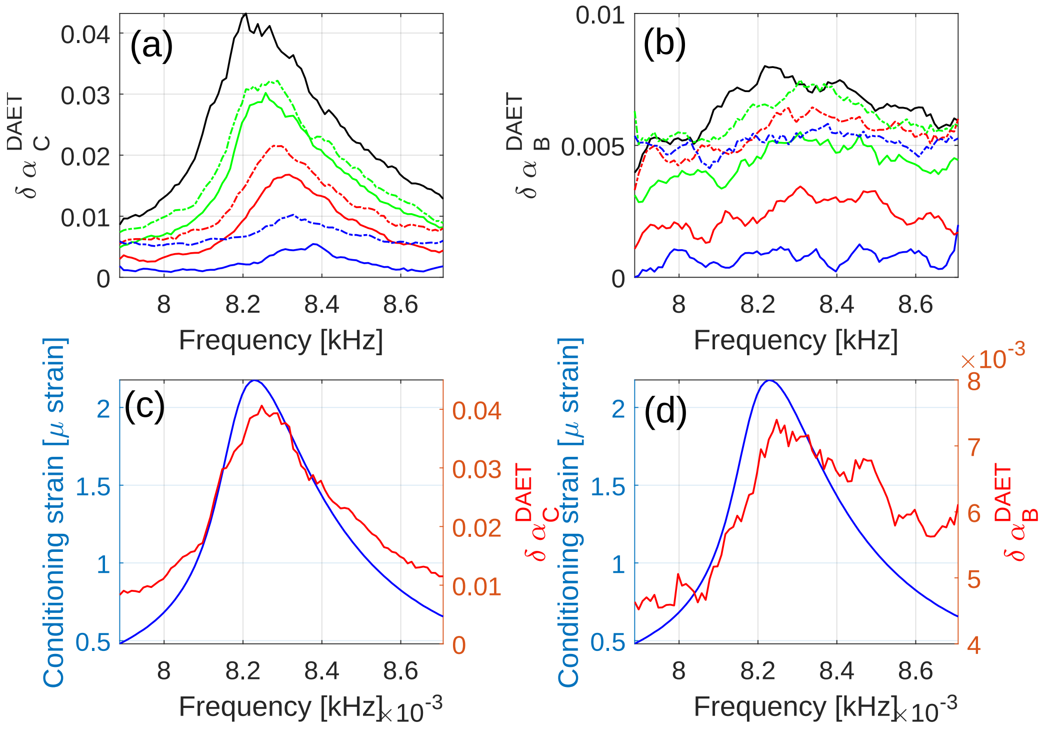

Figure A.2. Frequency dependence of damping variations.

Also for what concerns strain dependence (Fig. A.3), the behavior of damping is similar to that of velocity (Fig. 7). For DAET measurements, subtracting baseline data from conditioned data allows to remove the loop (pink symbols). NRUS measurements do not allow complete removal of hysteresis by subtraction and furthermore the strain dependence of damping looks qualitatively different respect to the one obtained using local (DAET) measurements.

Concluding the analysis of damping dependence, we remark that the peaks distortion due to fast and slow dynamics in NRUS measurement, must be considered to have a correct estimation of damping. In other words, the definition of Q-factor as the half-width of the resonance curve must be revisited.

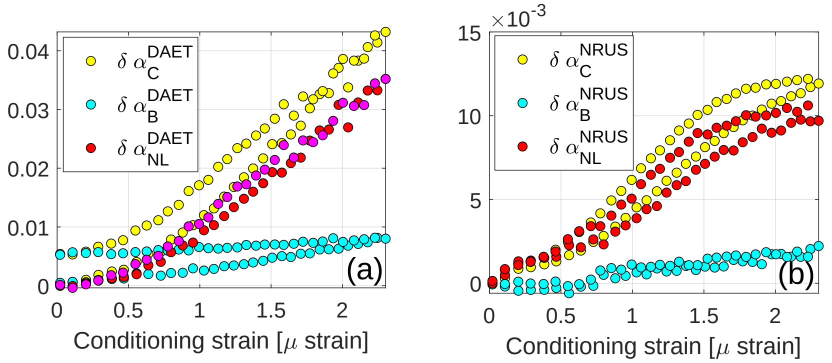

Figure A.3. Strain dependence of damping variations.

B Anisotropy

Let us assume we measure longitudinal/shear velocities in the same spatial position (in the center of the bar), but propagating in orthogonal directions (longitudinal δv_{11} and transverse δv_{21}, with respect to the conditioning loading). Anisotropy is defined as the linear correlation between them,

$$\delta v_{11} = k_a \delta v_{21}, \tag{B.1}$$

where k_a is the anisotropy coefficient and depends on the acoustoelastic coefficients and the Poisson effect.

In our study, we are considering longitudinal/shear waves in NRUS/DAET experiments, respectively. DAET measurements correspond to δv_{21}, measured in the center of the bar, while NRUS velocity measurements are averaging. As shown elsewhere[1], averaged values of velocity variations are proportional to velocity variation in the center, with a proportionality constant k_p which depends on the strain profile. It follows:

$$\begin{aligned} \delta v_{21} &= \delta v^{\mathrm{DAET}}, \\ \delta v_{11} &= k_p \delta v^{\mathrm{NRUS}}, \\ \delta v^{\mathrm{NRUS}} &= \frac{k_a}{k_\mathrm{p}} \delta v^{\mathrm{DAET}} = k' \delta v^{\mathrm{DAET}}. \end{aligned} \tag{B.2}$$

If k_p depends on strain as a consequence of the difference in strain profile at low and large strain amplitudes, it follows that the linear correlation expected from Eq. B.1 may not hold.

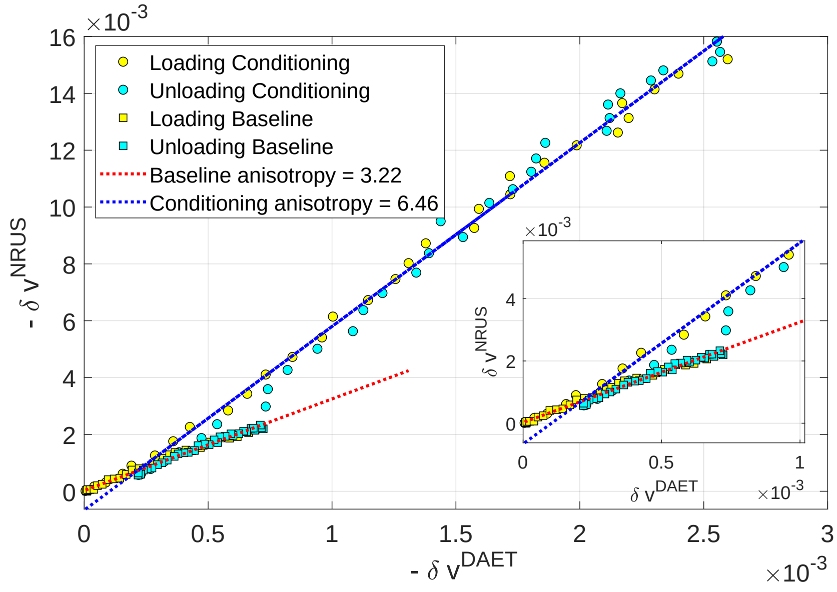

Figure B.1. Anisotropy.

In Fig. B.1, we discuss such correlation plotting NRUS vs. DAET measured velocities, both baseline and conditioned and during both loading and unloading. When we consider a low amplitude of excitation (baseline data), the linear correlation is preserved (see also the zoom in the inset). We obtain a corrected anisotropy coefficient $k' = 3.22$, in good agreement with that obtained for another Czech sandstone sample tested in different conditions, with low drive amplitude[1]. When drive amplitude increases (conditioned data), the behavior is not perfectly linear thus suggesting k_p to be strain dependent. Deviations from linearity are small and thus an anisotropy coefficient could be eventually calculated: $k' = 6.46$. This value is significantly different from that corresponding to baseline data.

C Reconstruction of velocity dependence from a frequency sweep at constant amplitude

The experiment performed consists in measuring wave velocity (using DAET) while conditioning the sample with sweeps in frequency increasing the conditioning amplitude. In the main text, for each sweep the maximum strain and the velocity variation detected at the resonance frequency were measured and their relation analysed (Fig. 7a). However, considering only data for one drive amplitude, each frequency of the sweep is probing the sample at a different strain level, defined by the amplitude of the resonance curve at that given frequency (see Fig. 5).

Therefore, a single drive amplitude might contain sufficient information to define the relation between strain and velocity variations. For a given A_i, for each ω_j a pair $(\varepsilon_C(i,j), \delta v_{C,B}^{DAET}(i,j))$ is measured. Using Eq. 9, we can derive the nonlinear contribution to velocity variation $\delta v_{NL}^{DAET}(i,j)$, which is plotted vs. $\varepsilon_C(i,j)$ for fixed i, in Fig. C.1a. We show results for a low (blue) and a high (red) conditioning amplitude. In the two conditions, the actual strain in the material at various frequencies span different regions (as resulting from the shape of the resonance curves). As visible, the two sweeps probe with good accuracy a different portion of the predicted strain dependence of velocity (solid dashed curve reported in the plot, taken from Fig. 7a).

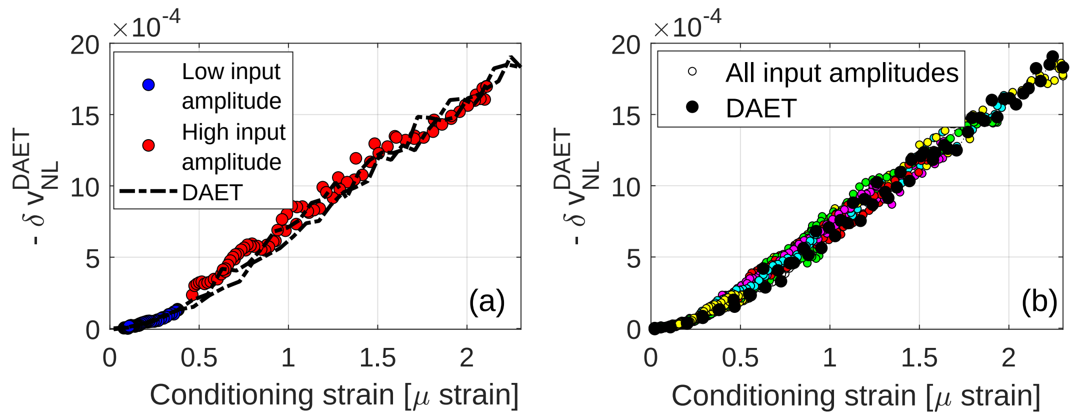

Figure C.1. Reconstruction of the corrected DAET relative velocity variation vs. strain amplitude derived from one sweep in frequency at constant input amplitude. (a) Data from low and high conditioning amplitudes; (b) data from all conditioning amplitudes (different colored symbols refer to measurements at different excitation amplitudes for all frequencies). In both subplots, results are compared with those obtained sweeping over different amplitudes (dashed black lines).

This is true for all amplitudes of excitation, as shown in subplot (b), where results for several conditioning amplitudes are reported. Each color is a different input amplitude and each point is a different frequency. All data fall on the same curve, exploring different strain ranges while increasing the drive amplitude, but describing the same dependence on strain. The dependence of the maximum relative velocity variation derived using a sequence of sweeps (as in the main text) is reported as symbols, with good agreement. In conclusion, as a novelty of the approach proposed here, we can claim that a sweep over frequency at a single conditioning amplitude can be used to describe reasonably well the dependence of velocity variations in the full (or in a proper) strain range.

References

1. Zeman, R., Kober, J., Nistri, F. & Scalerandi, M. Relaxation of Viscoelastic Properties of Sandstones: Hysteresis and Anisotropy. *Rock Mech. Rock Eng.* DOI: 10.1007/s00603-024-03914-6 (2024).
